# Supplementary material for: Practices and drivers of self-medication with antibiotics among undergraduate medical students in Eastern Uganda: A cross-sectional study
Source: PLoS One. 2023 Dec 21;18(12):e0293685. doi: 10.1371/journal.pone.0293685 (PMC10734914; doi:10.1371/journal.pone.0293685)
Supplement: S1 Checklist — (DOCX) [file pone.0293685.s001.docx]

STROBE Statement—checklist of items that should be included in reports of observational studies

|  | Item No. | Recommendation | Page  No. | Relevant text from manuscript |
| --- | --- | --- | --- | --- |
| **Title and abstract** | 1 | 1. Indicate the study’s design with a commonly used term in the title or the abstract   The title of the study contains the study design | 1 | A cross-sectional study |
|  |  | (*b*) Provide in the abstract an informative and balanced summary of what was done and what was found. The abstract is a balanced summary of the study aims, methods, results and conclusion | 1-2 |  |
| Introduction | | | |  |
| Background/rationale | 2 | Explain the scientific background and rationale for the investigation being reported  The background contains brief summary of the critical literature findings and provides a rationale for doing the study | 3-4 |  |
| Objectives | 3 | State specific objectives, including any prespecified hypotheses  The objective of the study is stated at the end of the background | 4 | The aim of conducting this study was to assess the practices and drivers of self-medication with antibiotics among university students at Busitema University, Mbale Campus in Eastern Uganda. |
| Methods | | | |  |
| Study design | 4 | Present key elements of study design early in the paper. This has been done | 4 |  |
| Setting | 5 | Describe the setting, locations, and relevant dates, including periods of recruitment, exposure, follow-up, and data collection  This has been addressed in the methods section | 4-6 | Setting: Busitema University, period of recruitment 28^th^ October-30^th^ November 2022, data collection: self-administered questionnaire |
| Participants | 6 | (*a*) *Cohort study*—Give the eligibility criteria, and the sources and methods of selection of participants. Describe methods of follow-up  *Case-control study*—Give the eligibility criteria, and the sources and methods of case ascertainment and control selection. Give the rationale for the choice of cases and controls  *Cross-sectional study*—Give the eligibility criteria, and the sources and methods of selection of participants. This has been described in the section for sampling method and procedure | 4 | Sampling method and procedure |
|  |  | (*b*) *Cohort study*—For matched studies, give matching criteria and number of exposed and unexposed  *Case-control study*—For matched studies, give matching criteria and the number of controls per case |  |  |
| Variables | 7 | Clearly define all outcomes, exposures, predictors, potential confounders, and effect modifiers. Give diagnostic criteria, if applicable  The outcomes for the study including the independent variables have been described in the section for the study variables | 5 |  |
| Data sources/ measurement | 8* | For each variable of interest, give sources of data and details of methods of assessment (measurement). Describe comparability of assessment methods if there is more than one group  This was described in the section of study variables and data collection procedure and tool | *5-6* |  |
| Bias | 9 | Describe any efforts to address potential sources of bias  Efforts were made to address potential sources of bias | 6 | The anonymized Kobo Collect tool minimized any potential bias, especially social desirability bias |
| Study size | 10 | Explain how the study size was arrived at  This was described in the section of the sampling procedure | 5 | The study sample size of 326 was arrived at using the Kish-Leslie formula |

Continued on next page

| Quantitative variables | 11 | Explain how quantitative variables were handled in the analyses. If applicable, describe which groupings were chosen and why  This was described in the section of the data analysis | 6 |  |
| --- | --- | --- | --- | --- |
| Statistical methods | 12 | 1. Describe all statistical methods, including those used to control for confounding   This was described in the section of the data analysis | 6 |  |
|  |  | (*b*) Describe any methods used to examine subgroups and interactions Not applicable | 6 |  |
|  |  | (*c*) Explain how missing data were addressed Not applicable | 6 |  |
|  |  | (*d*) *Cohort study*—If applicable, explain how loss to follow-up was addressed Not applicable  *Case-control study*—If applicable, explain how matching of cases and controls was addressed Not applicable  *Cross-sectional study*—If applicable, describe analytical methods taking account of sampling strategy This was described in the section of the data analysis | 6 |  |
|  |  | (*e*) Describe any sensitivity analyses Not applicable (NA) | 6 |  |
| Results | | | | |
| Participants | 13* | 1. Report numbers of individuals at each stage of study—eg numbers potentially eligible, examined for eligibility, confirmed eligible, included in the study, completing follow-up, and analysed   This was done | 7 | A total of 326 students at Busitema University Faculty of Health Sciences participated in the study |
|  |  | (b) Give reasons for non-participation at each stage This was not done |  |  |
|  |  | (c) Consider use of a flow diagram This was not done |  |  |
| Descriptive data | 14* | (a) Give characteristics of study participants (eg demographic, clinical, social) and information on exposures and potential confounders This was done (table 1) | 7-8 |  |
|  |  | (b) Indicate number of participants with missing data for each variable of interest No missing values |  |  |
|  |  | (c) *Cohort study*—Summarise follow-up time (eg, average and total amount) NA |  |  |
| Outcome data | 15* | *Cohort study*—Report numbers of outcome events or summary measures over time |  |  |
|  |  | *Case-control study—*Report numbers in each exposure category, or summary measures of exposure |  |  |
|  |  | *Cross-sectional study—*Report numbers of outcome events or summary measures  This was done | *9-10* |  |
| Main results | 16 | (*a*) Give unadjusted estimates and, if applicable, confounder-adjusted estimates and their precision (eg, 95% confidence interval). Make clear which confounders were adjusted for and why they were included NA |  |  |
|  |  | (*b*) Report category boundaries when continuous variables were categorized NA |  |  |
|  |  | (*c*) If relevant, consider translating estimates of relative risk into absolute risk for a meaningful time period NA |  |  |

Continued on next page

| Other analyses | 17 | Report other analyses done—eg analyses of subgroups and interactions, and sensitivity analyses  This was done | 10-11 |  |
| --- | --- | --- | --- | --- |
| Discussion | | | | |
| Key results | 18 | Summarise key results with reference to study objectives  This was done | 13 | ^The study sought to determine the prevalence and reasons for SMA among undergraduate students pursuing health programs. Although self-medication was deemed unacceptable, nearly all the respondents had used SMA. The common reasons for SMA were prior use of the antibiotic drug, a perception that the illness was minor, and notions that they were healthcare providers. The commonly used SMA was metronidazole and amoxicillin which were used for conditions such as diarrhea, peptic ulcer, and wound infections. Practices of inappropriate use of SMA were prevalent including multiple antibiotic use, switching to another antibiotic, and frequent SMA whenever they were sick.^ |
| Limitations | 19 | Discuss limitations of the study, taking into account sources of potential bias or imprecision. Discuss both direction and magnitude of any potential bias  This was done | 15 |  |
| Interpretation | 20 | Give a cautious overall interpretation of results considering objectives, limitations, multiplicity of analyses, results from similar studies, and other relevant evidence  This was done | 13-15 |  |
| Generalisability | 21 | Discuss the generalisability (external validity) of the study results  This was done | 15 |  |
| Other information | |  | | |
| Funding | 22 | Give the source of funding and the role of the funders for the present study and, if applicable, for the original study on which the present article is based  This has been included in the manuscript | 16 | Research reported in this publication was supported by the Fogarty International Center of the National Institutes of Health, U.S. Department of State's Office of the U.S. Global AIDS Coordinator and Health Diplomacy (S/GAC), and President's Emergency Plan for AIDS Relief (PEPFAR) under Award Number IR25TW011213. The funders had no role in study design, data collection and analysis, decision to publish, or preparation of the manuscript. The content is solely the responsibility of the authors and does not necessarily represent the official views of the National Institutes of Health. |

*Give information separately for cases and controls in case-control studies and, if applicable, for exposed and unexposed groups in cohort and cross-sectional studies.

**Note:** An Explanation and Elaboration article discusses each checklist item and gives methodological background and published examples of transparent reporting. The STROBE checklist is best used in conjunction with this article (freely available on the Web sites of PLoS Medicine at http://www.plosmedicine.org/, Annals of Internal Medicine at http://www.annals.org/, and Epidemiology at http://www.epidem.com/). Information on the STROBE Initiative is available at www.strobe-statement.org.
