# Supplementary material for: Practices and drivers of self-medication with antibiotics among undergraduate medical students in Eastern Uganda: A cross-sectional study
Source: PLoS One. 2023 Dec 21;18(12):e0293685. doi: 10.1371/journal.pone.0293685 (PMC10734914; doi:10.1371/journal.pone.0293685)
Supplement: Attachment — Submitted filename: Reviewers Comments Self Medication Uganda.docx [file pone.0293685.s003.docx]

REVIEWER’S COMMENTS

**MANUSCRIPT TITLE**: Practices and Drivers of Self-Medication with Antibiotics among Undergraduate Medical Students in Eastern Uganda: A Cross-sectional Study

**MANUSCRIPT ID**: PONE-D-23-21270

**Specific comments**

Page 3, Line 51: Ampiclox, Septrin: It is preferable to write the medications by their internationally recognized names or to use the trademark sign as superscript i.e. Septrin ®

Page 8, Table 1: Distance to health facility? - This question may be relevant if the study is community-based or the university is non-residential. If the university has a health facility, you may want to re-consider inclusion of the question.

Page 8, Table 1: Question – Are you a direct student? - For the sake of readers not from your region, kindly explain what is meant by direct and upgrading student

Table 2 is too extensive (almost two pages): The authors are advised to revise it either by changing part of its contents to charts and including some as statements in the text.

Page 9, Table 2: Question - Last time you self-treated? - For the sake of consistency, the authors are advised to stick to the term "self-medication"

Page 9, Table 2: Source of antibiotics Drug shop/clinc: This may confuse the readers - drug shop/clinic.

The authors may need to explain the difference using foot notes.

Page 10, Table 2: At home - I will suggest using leftovers from previous use" instead of "at home

Page 10, Table 2: Street: What is meant by this? Kindly clarify

Page 11, Table 2: Acceptable practice, good practice: How does one differentiate between "acceptable" and "good " practice?

Page 13, Line 155 : nearly all the respondents **had ever used** SMA: Please delete "ever". It gives a contrary meaning to the sentence.

Page 15, Line 215: Conclusion: Please summarize. There is no need to repeat most of the study findings here
