# Supplementary material for: Practices and drivers of self-medication with antibiotics among undergraduate medical students in Eastern Uganda: A cross-sectional study
Source: PLoS One. 2023 Dec 21;18(12):e0293685. doi: 10.1371/journal.pone.0293685 (PMC10734914; doi:10.1371/journal.pone.0293685)
Supplement: Attachment — Submitted filename: Response to Reviewers.docx [file pone.0293685.s004.docx]

REVIEWER’S COMMENTS

**MANUSCRIPT TITLE**: Practices and Drivers of Self-Medication with Antibiotics among Undergraduate Medical Students in Eastern Uganda: A Cross-sectional Study

**MANUSCRIPT ID**: PONE-D-23-21270

**Reviewer #1**

| **Comment** | **Response** | **Page** |
| --- | --- | --- |
| Reviewer #1: The authors have produced an useful piece of work on the the prevalence and factors associated with self-medication in Uganda. | We thank you for the positive appreciation of our work. |  |
| Abstract 1. The following statement was mentioned twice in the abstract: "The major drivers for self-medication were having a minor illness, prior experience with the drug, and the perception that they were already healthcare workers" | We thank you for the insightful observation regarding repeated statements in the abstract. We have taken note of the comment and revised it as per the guidance | Line 32-33  Page 2 |
| Background 1. Generic name need to be used for medications. | We thank you for the helpful suggestion to use generic names for medications. We have taken note of the comment and revised it accordingly. As per the guidance, generic names have now been used in the work. | Line 58-59  Page 2 |

**Reviewer #2**

| **Comment** | **Response** | **Page** |
| --- | --- | --- |
| Page 3, Line 51: Ampiclox, Septrin: It is preferable to write the medications by their internationally recognized names or to use the trademark sign as superscript i.e. Septrin ® | We thank you for pointing out this. We agree that medicines are written using their generic names. We have taken note of the comment and revised the naming of drugs to include generic names. | Page 3, line 58-59 |
| Page 8, Table 1: Distance to health facility? - This question may be relevant if the study is community-based or the university is non-residential. If the university has a health facility, you may want to re-consider inclusion of the question. | We thank you for the insightful observation. Although the university is non-residential, all students have access to the teaching hospital which situated within the residential location for most participants. We have considered the guidance to leave out this variable as it may not be relevant for this study population. | Page 8 table 1 |
| Page 8, Table 1: Question – Are you a direct student? - For the sake of readers not from your region, kindly explain what is meant by direct and upgrading student | We thank you for noticing the need to clarify this variable. We have taken note of the comment and revised this variable to mean the mode of university admission (diploma scheme or through high school) | Page 8  Table 1 |
| Table 2 is too extensive (almost two pages): The authors are advised to revise it either by changing part of its contents to charts and including some as statements in the text. | Thank you for the insightful observation. We agree table 2 was long. We have considered the guidance to include some statements as text which now has reduced the content of table 2 | Table 2  Page 10-11 |
| Page 9, Table 2: Question - Last time you self-treated? - For the sake of consistency, the authors are advised to stick to the term "self-medication" | We thank you for the comment to maintain consistency in the work. We have taken note of the comment and revised it accordingly. It is now captured as last time of self-medication | Table 2 |
| Page 9, Table 2: Source of antibiotics Drug shop/clinc: This may confuse the readers - drug shop/clinic. The authors may need to explain the difference using foot notes. | We thank you for the comment. We have modified the naming to make it more meaningful for the readers. As guided, we have added the footnotes that describe what a drug shop is in our setting. We have added drugs sold in the streets to the section of drug shop/clinic since operate in almost the way | Page 10  Table 2 |
| Page 10, Table 2: At home - I will suggest using leftovers from previous use" instead of "at home | We thank you for the suggestion to use the term leftovers from the previous use. We have revised it to ‘leftovers from previous use’ instead of ‘at home’ | Table 2 |
| Page 10, Table 2: Street: What is meant by this? Kindly clarify | We thank you for the comment. We have modified the variable to ‘roadside selling point’ and added the footnotes to describe what that means | Page 10  Table 2 |
| Page 11, Table 2: Acceptable practice, good practice: How does one differentiate between "acceptable" and "good " practice? | We thank you for the comment. We agree that it may be difficult to differentiate between ‘acceptable’ and ‘good’ practice. Because participants may have interpreted the two concepts synonymously, we have decided to combine the two. The variable is now worded as ‘acceptable/good/’ practice | Page 11  Table 2 |
| Page 13, Line 155 : nearly all the respondents **had ever used** SMA: Please delete "ever". It gives a contrary meaning to the sentence. | Thank you for the insightful observation. We agree the modifier gives a different meaning to it. We have revised the statement to make it clearer | Line 324  Page 13 |
| Page 15, Line 215: Conclusion: Please summarize. There is no need to repeat most of the study findings here | We thank you for the suggestion to summarize the conclusion. We have revised it as per the guidance. | Page 15  Line 391-400 |
