# Supplementary material for: Practices and drivers of self-medication with antibiotics among undergraduate medical students in Eastern Uganda: A cross-sectional study
Source: PLoS One. 2023 Dec 21;18(12):e0293685. doi: 10.1371/journal.pone.0293685 (PMC10734914; doi:10.1371/journal.pone.0293685)
Supplement: Attachment — Submitted filename: Response to Reviewers 09th Oct 2023.docx [file pone.0293685.s005.docx]

REVIEWER’S COMMENTS

**MANUSCRIPT TITLE**: Practices and Drivers of Self-Medication with Antibiotics among Undergraduate Medical Students in Eastern Uganda: A Cross-sectional Study

**MANUSCRIPT ID**: PONE-D-23-21270

**Reviewer #1**

| **Comment** | **Response** | **Page** |
| --- | --- | --- |
| Page 17, Lines 289-90: Please check the referencing - Organization WH - To be written as WHO (in full) | We thank you for the comment. We have revised it to now read as World Health Organization (WHO) | Page 17  Line 289 |
| Page 17: References nos 2-10 - Please check these references; names of journals are missing. This sometimes happens with the software being used for referencing.  Page 18, Reference no 20 - Same comment as above | We thank you for the insightful guidance. We have revised the entire reference list to include names of Journals. The referencing in the software were edited to include names of journals as per your guidance. | Page 17-18  Line 289-242 |
