# Supplementary material for: Practices and drivers of self-medication with antibiotics among undergraduate medical students in Eastern Uganda: A cross-sectional study
Source: PLoS One. 2023 Dec 21;18(12):e0293685. doi: 10.1371/journal.pone.0293685 (PMC10734914; doi:10.1371/journal.pone.0293685)
Supplement: Attachment — Submitted filename: Response to Reviewers 11th Oct 2023.docx [file pone.0293685.s006.docx]

REVIEWER’S COMMENTS

**MANUSCRIPT TITLE**: Practices and Drivers of Self-Medication with Antibiotics among Undergraduate Medical Students in Eastern Uganda: A Cross-sectional Study

**MANUSCRIPT ID**: PONE-D-23-21270

**Reviewer #1**

| **Comment** | **Response** | **Page** |
| --- | --- | --- |
| Thank you for updating the manuscript. However, there are some outstanding issues. This may be due to the reference manager used. I would advise doing some of the changes manually to ensure correctness. Please check the following: References 1-4, 8, 9,18 Please check reference 1 (Organization WH) to World Health Organization. | Thank you for identifying the missing journal names in the reference list. I have reviewed the reference list to ensure correctness Reference numbered has now been corrected from Organization WH to World Health Organization.  References 1-4, 8, 9 and 18 have journal names  Reference 1& 2: The Lancet Infectious Diseases  Reference 4: Plos One  Reference 8: Malaria Journal  Reference 9: Saudi Pharmaceutical Journal  Reference 18: Antibiotics | Page 17-18  Line 289-242 |
